# The Language, Tone and Prosody of Emotions: Neural Substrates and Dynamics of Spoken-Word Emotion Perception

**DOI:** 10.3389/fnins.2016.00506

**Published:** 2016-11-08

**Authors:** Einat Liebenthal, David A. Silbersweig, Emily Stern

**Affiliations:** ^1^Department of Psychiatry, Brigham and Women's HospitalBoston, MA, USA; ^2^Department of Radiology, Brigham and Women's HospitalBoston, MA, USA

**Keywords:** emotions, semantics, amygdala, word processing, fMRI, ERPs (event-related potentials), speech perception, voice perception

## Abstract

Rapid assessment of emotions is important for detecting and prioritizing salient input. Emotions are conveyed in spoken words via verbal and non-verbal channels that are mutually informative and unveil in parallel over time, but the neural dynamics and interactions of these processes are not well understood. In this paper, we review the literature on emotion perception in faces, written words, and voices, as a basis for understanding the functional organization of emotion perception in spoken words. The characteristics of visual and auditory routes to the amygdala—a subcortical center for emotion perception—are compared across these stimulus classes in terms of neural dynamics, hemispheric lateralization, and functionality. Converging results from neuroimaging, electrophysiological, and lesion studies suggest the existence of an afferent route to the amygdala and primary visual cortex for fast and subliminal processing of coarse emotional face cues. We suggest that a fast route to the amygdala may also function for brief non-verbal vocalizations (e.g., laugh, cry), in which emotional category is conveyed effectively by voice tone and intensity. However, emotional prosody which evolves on longer time scales and is conveyed by fine-grained spectral cues appears to be processed via a slower, indirect cortical route. For verbal emotional content, the bulk of current evidence, indicating predominant left lateralization of the amygdala response and timing of emotional effects attributable to speeded lexical access, is more consistent with an indirect cortical route to the amygdala. Top-down linguistic modulation may play an important role for prioritized perception of emotions in words. Understanding the neural dynamics and interactions of emotion and language perception is important for selecting potent stimuli and devising effective training and/or treatment approaches for the alleviation of emotional dysfunction across a range of neuropsychiatric states.

## Introduction

Spoken words naturally contain linguistic and paralinguistic elements that are both important and mutually informative for communication. The linguistic information consists of the literal, symbolic meaning of the word, whereas the paralinguistic information consists of the physical, contextual form of the word. For example, the meaning of the word “mad,” whether spoken in the sense of “mentally disturbed,” “furious,” or “wildly excited,” can be disambiguated based on evaluation of contextual paralinguistic information such as the speaker's current emotional status, as disclosed by their voice tone and facial expression. The linguistic and paralinguistic bits of information unveil in parallel as the spoken word unfolds over time. However, the neural dynamics of each process and the nature of neural interactions between linguistic and paralinguistic processes in spoken word perception are not well understood.

In this paper, we review the literature on perception of emotion in faces, written words, and voices, as a basis for understanding the neural architecture of emotion perception in spoken words. In particular, we critically consider evidence from animal, and human lesion and neuroimaging, studies for the existence of a fast route for emotion perception in spoken words that is analogous to the route described for facial expressions. We compare the characteristics of auditory and visual routes to the amygdala, in terms of neural dynamics, hemispheric lateralization, and functionality, across these stimulus classes. The comparison of neural substrates and neural dynamics of emotion perception across sensory modalities (auditory, visual) and stimulus types (non-verbal, verbal) informs the issue of whether certain aspects of the neural processing of emotions can be considered supramodal and universal, and therefore broadly applicable to linguistic input. We base the initial inquiry on the perception of emotion in faces because current neural models (Vuilleumier et al., 2003; Johnson, 2005) make detailed predictions regarding the neural underpinnings of fast and slow responses. We then consider intermediate stimuli that share additional characteristics with spoken words (specifically, written words are also linguistic, and nonverbal sounds are also auditory). We also draw a comparison between the spatial cues of visual stimuli and the temporal cues of auditory stimuli, which convey dominantly emotional paralinguistic or linguistic information depending on their frequency.

**Figure 1:**
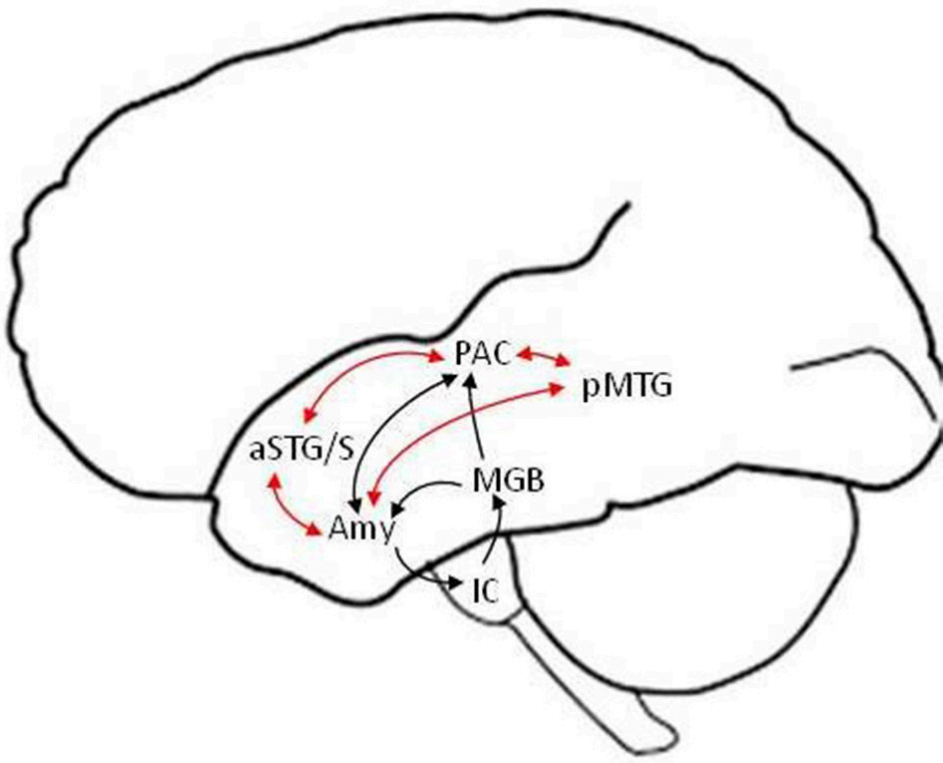
**Schematic model of putative fast (black arrows) and slow (red arrows) subcortical and temporal lobe pathways in the left hemisphere for the perception of emotional speech**. The detection of basic emotional categories (e.g., joy, sadness) from brief and salient non-verbal utterances (e.g., laugh, cry) is suggested to be mediated by fast routes that bypass non-primary cortical areas and reach the amygdala within ~120 ms. The detailed evaluation of emotions based on the meaning and prosody of verbal utterances is suggested to involve slower efferent projections from non-primary auditory (e.g., aSTG/S voice) and language association (e.g., pMTG semantic) areas to the amygdala. Similar structural pathways are predicted in the right hemisphere (not shown), with differences in the strength of functional connections, including from higher-order association cortical areas, potentially accounting for differences in hemispheric lateralization between fast and slow pathways (see text for details). Efferent connections from cortical to subcortical areas (other than the amygdala) and output connections from the amygdala to paralimbic cortices and the hippocampus are not depicted, for clarity. IC, inferior colliculus; MGB, medial geniculate body; Amy, amygdala; PAC, primary auditory cortex; aSTG/S, anterior superior temporal gyrus and sulcus; pMTG, posterior middle temporal gyrus.

We discuss the neural underpinnings of emotion perception within the framework of a “valence-general” hypothesis, according to which the perception of both positive and negative valences is realized by flexible neuronal assemblies in limbic and paralimbic brain regions (Barrett and Bliss-Moreau, 2009; Lindquist et al., 2016). In this framework, arousal (i.e., the degree of emotional salience) and not valence (i.e., degree of positive or negative emotional association) is the dominant variable according to which the level of activation in different neuronal assemblies varies. Another point of emphasis is that language provides the context for experiencing and understanding emotions (and the world in general) (Barrett et al., 2007). Thus, our neural model (depicted schematically in Figure 1) presumes that the perception of emotional speech is a product of neural interactions between limbic and paralimbic emotional, cortical auditory and semantic, as well as frontal cognitive control areas. The amygdala is thought to play a central role at the intersection of these networks, as a fast salience detector alerting limbic, paralimbic, endocrine, and autonomic nervous systems to highly arousing stimuli. But the amygdala is also involved in slower evaluation of stimulus valence and arousal, interactively with associative cortical networks. Primary evidence for the existence of fast direct, and slow indirect via non-primary cortical, routes for emotion perception comes from electrophysiological studies probing neural activity with high temporal resolution, as well as focal lesion studies of patients with focal subcortical or cortical lesions.

Face perception is thought to rely on both a fast neural pathway specialized for gross analysis of emotional expression, and a slower neural pathway for identity recognition and detailed evaluation of emotional expression. Evidence for different hemispheric lateralization of the amygdala is consistent with the possibility of a separation of neuroanatomical pathways for slow (left hemisphere dominance) and fast (right hemisphere dominance) processes underlying face perception (Morris et al., 1999; Wright et al., 2001). Electrophysiological studies of face perception suggest that early (<120 ms) differential responses to emotional expressions reflect the activity of fast direct routes, whereas later (>120 ms) differential responses to emotional expressions reflect the activity of indirect routes via non-primary visual cortex (Noesselt et al., 2002; Pourtois et al., 2004; West et al., 2011). This alleged division of labor for face emotion and identity perception has been related to behavioral findings that low-spatial frequency global configurational cues are sufficient to convey coarse face emotional expressions, whereas high-spatial frequency fine-grained cues are needed to convey precise face identity features (Costen et al., 1996; Liu et al., 2000). Emotional aspects of stimuli are important for determining the level of significance and prioritizing time-sensitive salient input potentially critical for survival. Thus, an evolutionary advantage to faster processing of emotional input may have contributed to a differentiation of neural pathways for low and high spatial frequency cues. According to this theoretical framework, a direct pathway for extraction of low spatial frequencies evolved that can provide fast, subconscious appraisal of stimuli important for survival and for non-verbal communication (Vuilleumier et al., 2003; Johnson, 2005). Drawing on the findings in the visual modality, and recognizing that the timing of neural processing may depend on a variety of factors such as the sensory modality (e.g., basic auditory processing may be faster than basic visual processing), the stimulus complexity (e.g., linguistic stimuli may be processed more slowly than non-linguistic stimuli), and the stimulus category (e.g., some categories such as faces may confer special processing advantages), our working hypothesis is that neural processing within the first ~120 ms from stimulus presentation could be related to the activation of fast routes for prioritized processing.

Whether a processing advantage similar to that observed for emotional faces also extends to symbolic input such as written words, is controversial. While evidence exists for differential processing of emotional words early in the processing chain and subliminally (Gaillard et al., 2006; Kissler and Herbert, 2013), it remains unclear through what neural pathway/s. Compared to the perception of facial expressions which is acquired early in development and is perhaps even innate (Johnson, 2005), language comprehension is a learned skill that develops later. Thus, top-down modulation by semantic cortical networks and contextual learning have been suggested to play an important role in mediating prioritized emotional word perception (Barrett et al., 2007).

In the auditory system, the voice parallels the face in that it conveys a person's identity and current emotional status. Some aspects of voice emotions (in particular emotional category, e.g., anger, disgust, fear, sadness, joy) are thought to be perceived quickly based on coarse tone and intensity analysis of brief segments of familiar non-verbal vocalizations (e.g., shriek, cry, laugh etc…), and may be mediated by a fast direct route. However, other aspects of voice emotions (in particular emotional prosody), and identity recognition, may require sampling of longer voice segments and a more detailed spectral analysis thereof, and may involve slower routes via non-primary cortical areas.

The voice is also the natural carrier of speech. The voice paralinguistic and linguistic cues are separated such that the low-frequency band primarily carries prosodic cues important for communication of emotions, whereas the high-frequency band primarily carries phonemic cues critical for verbal communication (Remez et al., 1981; Scherer, 1986). Neural processing of the spectrally slow-varying emotional prosody cues appears to involve more anterior auditory cortical areas in the superior temporal lobe than the processing of spectrally fast-varying phonemic cues (Belin et al., 2004; Liebenthal et al., 2005). Neural processing of emotional voice cues is also thought to involve auditory cortical areas predominantly in the right hemisphere, whereas that of phonemic cues predominantly auditory areas in the left hemisphere (Kotz et al., 2006; Scott and McGettigan, 2013). Which voice emotional cues confer a processing advantage, and through what neural routes, is an ongoing topic of investigation.

We conclude the paper with open questions that should be addressed in future research. In particular, the neural dynamics of direct and indirect routes for processing of different emotional cues require further study. For example, while both the amygdala and auditory cortices show sensitivity to various voice emotional cues, it remains unclear whether and under what circumstances, observed activation patterns are driven by the amygdala, are a result of cortical feedback connections to the amygdala, or both.

## Perception of face emotional expressions

Various behavioral observations suggest that emotional stimuli are more likely to draw attention and be remembered than neutral stimuli, and that the emotional modulation of perception and memory is involuntary (Anderson, 2005; Phelps and LeDoux, 2005; Vuilleumier, 2005). For example, emotional faces are more readily detected than neutral faces in visual search (Eastwood et al., 2001; Fox, 2002) and spatial orienting (Pourtois et al., 2005) tasks. Face emotional expressions can be conveyed by coarse cues: low-spatial frequency cues (2–8 cycles/face) are important for processing visual input in the periphery, at a distance, or in motion (Livingstone and Hubel, 1988; Merigan and Maunsell, 1993), and may aid in the perception of threat. For example, the general outline of the eyes (e.g., degree of widening, coarse gaze direction) is visible at low spatial frequency, and can contribute to determining a person's emotional status (Whalen et al., 2004). Low frequency cues also carry crude facial information (face configuration, emotional expression), which can be perceived by newborn infants in the absence of a mature visual cortex (Johnson, 2005). This is in contrast to the high-spatial frequency cues (8–16 cycles/face) that are important for analysis of the visual shape and texture underlying accurate face identification (Fiorentini et al., 1983; Liu et al., 2000).

The degree of salience of emotional faces has been found to be positively related to level of activity in the amygdala and occipito-temporal visual cortex including the fusiform gyrus (Adolphs et al., 1998; Morris et al., 1998; Vuilleumier et al., 2001b; Pessoa et al., 2002). The amygdala is thought to play a key role in evaluating the significance and arousal associated with, and mediating automatic responses to, emotional stimuli (LeDoux, 2000; Sander et al., 2003a; Phelps and LeDoux, 2005), through its rich input and output connections to many subcortical and cortical regions (Amaral et al., 2003). Damage to the amygdala has been shown to eliminate the enhanced response in visual cortex for emotional faces (Vuilleumier et al., 2004), although there are also contrary findings (Adolphs et al., 2005; Pessoa and Adolphs, 2010). Furthermore, emotional modulation of the neural response to faces arises very early, consistent with subcortical processing possibly at a subliminal level (Pourtois et al., 2005; Eimer and Holmes, 2007). To explain the automatic processing advantage of emotional faces, it has been proposed that coarse visual information relevant to emotional state (e.g., extent of eyes opening Whalen et al., 2004) is processed quickly via the amygdala and fed to visual cortex to enhance the processing of emotional information (Vuilleumier et al., 2003; Vuilleumier, 2005). Several lines of evidence, outlined below, support the existence of a direct subcortical pathway that mediates emotional influences on sensory processing (in parallel with the attentional modulation of sensory processing by frontal-parietal systems).

First, distinct patterns of spatial frequency sensitivity have been demonstrated in the fusiform cortex and the amygdala in several studies using functional magnetic resonance imaging (fMRI) or positron emission tomography (PET) neuroimaging, consistent with the idea that distinct neural pathways operate on different subsets of cues available concurrently in face images (Vuilleumier et al., 2003; Winston et al., 2003). The fusiform cortex is more responsive to fine-grained high-spatial frequency information, whereas the amygdala is selectively modulated by coarse low-spatial frequency information. In extensive fusiform areas, neural adaptation was observed to repetition of low- after high- spatial frequency stimuli but not the reverse, suggesting that only the high frequency input established a long lasting representation in this area (Vuilleumier et al., 2003). The fusiform receives major inputs from parvocellular channels with fine resolution but slow processing (Livingstone and Hubel, 1988; Merigan and Maunsell, 1993), and this area is known to be important for fine visual shape and texture analysis and face recognition (Kanwisher et al., 1997; Vuilleumier et al., 2001a). In contrast, the amygdala receives major inputs from magnocellular channels with coarse resolution but fast processing, through a retinal—superior colliculus—pulvinar subcortical pathway (Schiller et al., 1979; Livingstone and Hubel, 1988; Merigan and Maunsell, 1993). This latter pathway is thought to bypass the slower cortical processing in the ventral visual pathway, and enable crude but fast processing of fear-related (LeDoux, 1996; Morris et al., 1999, de Gelder et al., 2003) and more generally emotion-related (Zald, 2003), aspects of visual input that determine stimulus salience.

A second line of evidence comes from electrophysiological (electro- and magneto- encephalography) studies that delineate the temporal course of neural processing of emotional input. An early differential event-related potential (ERP) response to emotional versus neutral faces (Eimer and Holmes, 2002; Eger et al., 2003; Streit et al., 2003; Pourtois et al., 2004, 2005; van Heijnsbergen et al., 2007; Rudrauf et al., 2008; Rotshtein et al., 2010) is observed around 120 ms latency, earlier than the ERP response associated with face recognition around 170–200 ms (Bentin et al., 1996). This differential ERP response to emotional faces is preserved even when the stimuli are filtered to include only low spatial frequencies (Pourtois et al., 2005). In the visual modality, the time around 120 ms from stimulus presentation corresponds to the visual P1 response associated with pre-attentional perceptual processing (Di Russo et al., 2002, 2003; Liddell et al., 2004). The visual P1 is thought to be generated primarily in posterior occipito-temporal areas (Di Russo et al., 2002). However, amygdala responses to emotional stimuli demonstrated with intracranial recording within the same time range (Oya et al., 2002; Gothard et al., 2007), as well as findings of a diminished P1 response to emotional stimuli in patients with amygdala lesions (Rotshtein et al., 2010), are consistent with the possibility that neural generators in the amygdala also contribute to the P1 either directly or via modulation of cortical generators (Pourtois et al., 2013). In addition, earlier (<100 ms) responses to fearful versus happy faces have been recorded and localized to primary visual cortex (Noesselt et al., 2002; Pourtois et al., 2004; West et al., 2011), consistent with fMRI activation of this area by emotional faces (Vuilleumier et al., 2001b; Pessoa et al., 2002). The emotional enhancement of primary visual cortex at a time window preceding attentional enhancement is consistent with emotional modulation via a fast subcortical route. Overall, these findings are at the basis of models of visual perception placing the effects of prioritized processing of salient emotional material in the time range of about 120 ms from stimulus presentation (Vuilleumier et al., 2005).

Third, studies in patients with brain damage support the role of the amygdala in processing emotional cues in faces. Patients with extensive damage to the visual cortex resulting in hemispatial neglect, blindsight, or prosopagnosia, have been found to have residual ability for detection of faces and facial expressions (Morris et al., 2001; de Gelder et al., 2003; Pegna et al., 2005), suggesting that these functions can be accomplished subcortically. Patients with damage to the amygdala did not demonstrate the enhanced response in visual cortex for emotional faces (Vuilleumier et al., 2004). Furthermore, damage to the amygdala resulted in diminished early (100–150 ms) intracranial ERPs to fearful faces, consistent with a causal role for the amygdala in mediating the emotional enhancement of extrastriate visual cortex activity (Rotshtein et al., 2010). The amygdala response has also been shown to be modulated during subliminal processing of emotional faces (Whalen et al., 1998; Morris et al., 1999).

Finally, some evidence suggests that hemispheric lateralization may also differ between the slow cortical and fast subcortical face processing routes. In the amygdala, neural dynamics have been found to differ between the hemispheres such that the duration of response is shorter and the rate of adaptation is higher in the right compared to the left amygdala (Wright et al., 2001; Gläscher et al., 2004; Costafreda et al., 2008; Sergerie et al., 2008). Subliminal emotional stimuli activate predominantly the right amygdala (Morris et al., 1998; Gläscher and Adolphs, 2003; Pegna et al., 2005), whereas emotional information conveyed exclusively through language activates predominantly the left amygdala (Phelps et al., 2001; Olsson and Phelps, 2004). Taken together, these findings indicate the possibility that the right and let amygdala have somewhat different functions. Specifically, the right amygdala may play a primary role as an emotion detector, responding fast and at a subconscious level, possibly through the subcortical superior colliculus-pulvinar-amygdala pathway (LeDoux et al., 1984; Morris et al., 1999). In contrast, the left amygdala may play a primary role in evaluating the significance of emotional stimuli, responding more slowly, possibly through an indirect, cortical route (Vuilleumier et al., 2003; Winston et al., 2003).

In summary, results from neuroimaging, electrophysiological, and lesion studies support the existence of a subcortical route for fast and subliminal processing of coarse emotional face cues. This route is thought to mediate the sensory processing, and attentional and memory enhancements observed for emotional faces. It is important to note however, that various findings in the literature are consistent with the existence of multiple parallel routes for visual emotional processing that may also contribute to rapid processing of salient information and may not involve the amygdala. Anatomically, “shortcut” connections within inferotemporal cortex and from the lateral geniculate nucleus to extrastriate visual cortex have been demonstrated in the monkey (Felleman and Van Essen, 1991) and could contribute to rapid visual processing. Pessoa and Adolphs (2010) use the finding of a patient with bilateral amygdala lesions who is able to normally process fearful faces (Adolphs et al., 2005; Tsuchiya et al., 2009) as evidence suggesting that the amygdala is not essential for exhibiting an emotional processing advantage. In a magnetoencephalography study, Rudrauf et al. (2008) demonstrate that the temporal course of processing arousing visual information is most accurately predicted by two-pathway models which include additional parallel shortcut pathways reaching the amygdala, temporal pole and orbitofrontal cortex more directly, either via cortical-cortical long-range fasciculi or via subcortical routes. While these studies do not negate the existence of a rapid amygdala route, they are consistent with the idea that there may be additional routes that mediate the prioritization of emotional stimuli.

## Perception of emotional written-words

Compared to non-linguistic stimuli such as faces, words can convey emotional states with greater accuracy and finer nuances. The emotional content of written words can systematically and continuously be deconstructed along several primary dimensions, and in particular valence (degree of positive or negative emotional association) and arousal (degree of emotional salience), that are separable but interact (Bradley and Lang, 1999; Warriner et al., 2013). The question of whether an expedited subcortical route exists for visual processing of symbolic, detailed emotional input such as written words is contentious (Naccache and Dehaene, 2001; Gaillard et al., 2006). Semantic processing of words is associated with activity across extensive cortical networks (Binder and Desai, 2011), but it is unclear whether some level of analysis related to emotional content is accomplished subcortically. Observations that compared to neutral words, emotional words are more likely to be attended (Williams et al., 1996; Mogg et al., 1997; Anderson and Phelps, 2001), are better remembered (Kensinger and Corkin, 2004; Krolak-Salmon et al., 2004; Strange and Dolan, 2004; Vuilleumier et al., 2004; Kissler et al., 2006), and are also more quickly detected in a lexical decision task (Kanske and Kotz, 2007; Kousta et al., 2009; Scott et al., 2009; Vigliocco et al., 2014), have led to the suggestion that analysis of some emotional linguistic content (in particular, salience and emotional category) could be facilitated at a subcortical level. Connections from the amygdala to visual cortex (Amaral et al., 2003) and to the orbitofrontal cortex (Timbie and Barbas, 2015) could mediate the enhanced cortical processing of emotional words detected subliminally in the amygdala.

Greater activation of the amygdala for negative and positive valenced words relative to neutral words has been demonstrated with fMRI and PET in normal control subjects (Isenberg et al., 1999; Hamann and Mao, 2002; Kensinger and Schacter, 2006; Goldstein et al., 2007; Weisholtz et al., 2015). The level of activity in the amygdala was found to vary mostly with the level of word arousal, whereas activity in the orbitofrontal and subgenual cingulate cortex varied mostly with word valence (Lewis et al., 2007; Posner et al., 2009; Colibazzi et al., 2010), consistent with the hypothesized role of the amygdala as an emotional salience detector.

However, in general, language stimuli are less likely to activate the amygdala, particularly in the right hemisphere (Anderson and Phelps, 2001; Phelps et al., 2001; Olsson and Phelps, 2004; Goldstein et al., 2007; Costafreda et al., 2008). The weak response of the amygdala to language has been related to its reduced involvement in language processing, or even its inhibition by prefrontal cortex (Bechara et al., 1995; Rosenkranz et al., 2003; Pezawas et al., 2005; Blair et al., 2007). Another contributing factor could be that the amygdala response to language is highly dependent on the subjective relevance of words, and is therefore difficult to reliably detect across a group of individuals. Support for a high sensitivity of the amygdala response to individual variation in word processing comes from studies of patients with anxiety disorders. For example, elevated left amygdala activation and abnormal patterns of sensitization and habituation were observed in post-traumatic stress disorder (PTSD) relative to normal control subjects for trauma-related negative, but not panic-related negative, versus neutral written words (Protopopescu et al., 2005). Sensitivity of the amygdala response to individual variation is also demonstrated by a dependence on moment-by-moment subjective evaluation of emotional intensity and subsequent memory of stimuli (Canli et al., 2000; Protopopescu et al., 2005). A left amygdala preference for language could be due to the general dominance of the left hemisphere for language. Increased activation of the left amygdala for language could reflect increased functional connectivity with highly left-lateralized, higher-order semantic memory networks distributed across the temporal, parietal and frontal cortex (Binder and Desai, 2011). Effects of word frequency have been reported in the left amygdala (Nakic et al., 2006), also consistent with a linguistic basis for the lateralization pattern in this area for words. However, whether there exists a right amygdala advantage in a fast subcortical afferent route for subliminal processing of salient emotional words remains an entirely open question.

In terms of temporal course, emotionally arousing (positive and negative) relative to neutral words have most commonly been found to elicit a differential ERP response around 180–300 ms (Kissler et al., 2006; Thomas et al., 2007; Herbert et al., 2008; Schacht and Sommer, 2009; Scott et al., 2009; Hinojosa et al., 2010; Citron et al., 2011). The timing of the differential response to emotional written words is consistent with the timing of lexical access to written words (Schendan et al., 1998; Cohen et al., 2000b; Grossi and Coch, 2005) localized to the fusiform gyrus (Kissler et al., 2007; Schacht and Sommer, 2009). Lexical access occurs earlier for emotional (~220–250 ms) versus neutral (~320 ms) words (Kissler and Herbert, 2013), consistent with the behavioral enhancement of emotional words in lexical decision tasks. Earlier (80–180 ms) effects of arousal have been reported for highly familiar emotional words (Ortigue et al., 2004; Hofmann et al., 2009; Scott et al., 2009), and in individuals with elevated anxiety (Pauli et al., 2005; Li et al., 2007; Sass et al., 2010). These early effects are thought to reflect enhanced orthographic processing (Hauk et al., 2006), speeded lexical access (Hofmann et al., 2009), and even rudimentary semantic analysis (Skrandies, 1998), of high-frequency emotional words. Repeated association (i.e., contextual learning) of the visual orthographic form of the word with its emotional meaning may facilitate the processing of high-frequency emotional written words (Fritsch and Kuchinke, 2013).

Taken together, these findings suggest that the role of the amygdala in detecting and prioritizing time-sensitive salient input extends to written words. The bulk of current evidence, indicating predominant left lateralization of the amygdala response to words, and timing of emotional word effects attributable to speeded lexical access in extrastriate cortex, appears more consistent with an indirect cortical route to the amygdala than a direct route akin to that described for emotional faces. Nevertheless, faster afferent access to the amygdala may exist for specific words that are highly-familiar and highly emotionally-salient. Because the emotional relevance of words likely varies widely between individuals, this may lead to mixed or weak findings within and across studies.

## Perception of emotional non-verbal vocalizations

The voice is a particularly important medium for conveying emotional state because it is relatively independent of the listener's distance from, and ability to view, the speaker (unlike face cues). The acoustic cues conveying voice emotion—consisting of pitch (fundamental frequency), loudness (intensity), rhythm (duration of segments and pauses), and timbre (distribution of spectral energy) (Banse and Scherer, 1996; Grandjean et al., 2006)—are modulated by physiological factors (e.g., heart rate, blood flow, muscle tension) that vary as a function of a person's emotional state. Two main aspects of the voice are thought to convey emotional state on different time scales. The prosody of speech (discussed in the next section), consisting of pitch, loudness contour, and rhythm of speech articulation, evolves relatively slowly over suprasegmental speech intonations (>200 ms). The quality of non-speech vocalization (discussed in this section), consisting of timbre and abrupt, aperiodic spectral changes, emerges more rapidly (Pell et al., 2015), and has been shown to convey certain emotional categories (e.g., fear, disgust) potently (Banse and Scherer, 1996; Scott et al., 1997). Similar to emotional faces, emotional voices appear to confer perceptual advantages, as evidenced by improved memory for emotional over neutral nonspeech vocalizations (Armony et al., 2007) and priming effects across non-verbal vocalizations and faces or words conveying the same emotional category (Carroll and Young, 2005).

Similar to the increased activity observed in visual occipito-temporal cortex for emotional faces, emotional non-verbal vocalizations (e.g., scream, cry, laugh) produce increased activity in the auditory superior temporal cortex and the amygdala (Phillips et al., 1998; Morris et al., 1999; Sander and Scheich, 2001; Fecteau et al., 2007), albeit with a variable level and lateralization pattern in the amygdala. The mixed amygdala response to emotional vocalizations could be related to variations in the subjective level of arousal elicited by vocal stimuli (Schirmer et al., 2008; Leitman et al., 2010). The amygdala may be particularly responsive to short, nonverbal emotional vocalizations (Sander et al., 2003b; Fecteau et al., 2007; Frühholz et al., 2014) because they tend to carry higher emotional weight and be more emotionally salient than speech prosody which evolves over a longer suprasegmental time scale. The amygdala may also be activated particularly during implicit processing of vocal emotions (Sander et al., 2005; Bach et al., 2008; Frühholz et al., 2012). Rising sound intensity has been proposed as an elementary auditory warning cue (Neuhoff, 1998), and has been demonstrated to activate the right amygdala more than a comparable decline in sound intensity (Bach et al., 2008). This finding is compatible with findings in the visual modality associating the amygdala with emotional intensity detection (Bonnet et al., 2015), and more generally with emotional relevance detection (Sander et al., 2003a).

In terms of neural temporal course, ERP studies show that emotional non-verbal vocalizations are distinguished from neutral vocalizations as early as 150 ms after sound onset (Sauter and Eimer, 2010). In the auditory modality, this timing corresponds to obligatory processing of acoustic cues (e.g., pitch, intensity) in auditory cortex (Vaughan and Ritter, 1970; Näätanen and Picton, 1987), and has been linked to subliminal emotional salience detection based on integration of acoustic cues signaling the emotional significance of a sound (Paulmann and Kotz, 2008). The timing of these voice emotional effects is similar to the emotional effects seen in face perception (~120 ms), and this raises the possibility that attentional modulation of emotional voices and faces is mediated by common supramodal neural routes (Sauter and Eimer, 2010). A few studies have also reported earlier (in the 100 ms range) effects of emotions on vocalization perception. Interactions between sensory modality (auditory, visual, audiovisual) and valence (fear, anger, neutral) were seen on the amplitude of the N100 ERP response (Jessen and Kotz, 2011). Another study showed that affective (positive and negative) auditory conditioning modulated the magnetic ERP response to brief tones in the time range <100 ms, reflecting the activity of auditory sensory, frontal, and parietal cortex regions suggested to be part of an auditory attention network (Bröckelmann et al., 2011). Overall, these findings are consistent with the possibility of emotional enhancement of vocalization perception via rapid auditory pathways. Experimentally, early enhancement may be limited to conditions in which the input is very familiar (e.g., due to a small stimulus set, or conditioning).

Animal studies show that many neurons in the amygdala respond to broad-band sounds, with some neurons tuned to specific frequency bands, albeit not as narrowly as, and at a higher response threshold than, neurons in the tonotopically organized leminiscal pathway from medial geniculate body to auditory cortex (Bordi and LeDoux, 1992). A large proportion of amygdala neurons responding to sounds also exhibit high habituation rates (Bordi and LeDoux, 1992). The amygdala receives fast, direct auditory thalamic input from extraleminiscal areas that are one synapse away from the amygdala and weakly encode sound spectral properties. The amygdala also receives slow, indirect auditory cortical input from association areas that are several synapses removed from the amygdala and encode more detailed acoustic patterns of sounds (Bordi and LeDoux, 1992; LeDoux, 2000). The direct thalamic pathway to the amygdala could be important for fast, subliminal detection and evaluation of emotional cues in short vocalizations based on coarse spectral properties (LeDoux, 2000; Frühholz et al., 2014). Indeed, a recent neuroimaging study in humans found that amygdala activation is sensitive to voice fundamental frequency and intensity variations relevant to emotional state in short nonword utterances (Frühholz et al., 2012). On the other hand, emotional prosody in longer speech segments may be evaluated on longer time scales (Pell and Kotz, 2011) via an indirect cortical route to the amygdala (Frühholz et al., 2014).

In summary, the comparatively small body of work investigating the neural basis of voice perception indicates the possibility of a fast route for prioritized perception of emotional non-verbal vocalizations. This route appears to be responsive particularly to brief vocalizations in which emotions are conveyed categorically by voice tone and intensity. However, the precise physical and perceptual attributes of vocalizations potentially processed via a direct route to the amygdala, and the degree of overlap with neural processing described for emotional faces, require further study.

## Perception of emotional spoken words

Compared to written words, spoken words contain additional non-verbal emotional information (i.e., emotional prosody) that is physically and perceptually intertwined with the verbal information (Kotz and Paulmann, 2007; Pell and Kotz, 2011). The verbal and emotional cues in speech differ in their spectrotemporal properties. The phonemic cues consist primarily of relatively fast spectral changes occurring within 50 ms speech segments, whereas the prosodic cues consist of slower spectral changes occurring over more than 200 ms speech segments (syllabic and suprasegmental range). Emotional speech confers processing advantages such as improved intelligibility in noise background as well as faster repetition time for words spoken with congruent emotional prosody (Nygaard and Queen, 2008; Gordon and Hibberts, 2011; Dupuis and Pichora-Fuller, 2014).

Similar to brief emotional non-verbal vocalizations, emotional prosody in speech and speech-like sounds produces increased activity in the auditory superior temporal cortex (Grandjean et al., 2005; Sander et al., 2005; Beaucousin et al., 2007; Ethofer et al., 2009) and less consistently, in the amygdala (Wildgruber et al., 2005; Wiethoff et al., 2008). The amygdala is more likely to be activated by concurrent and congruent face and voice emotional cues than by emotional voices alone (Ethofer et al., 2006; Kreifelts et al., 2010). Damage to the amygdala has also only inconsistently been associated with impaired perception of emotion in voices (Scott et al., 1997; Anderson and Phelps, 1998; Sprengelmeyer et al., 1999; Adolphs et al., 2005). A recent fMRI study showed that damage to the left, but not the right, amygdala resulted in reduced cortical processing of speech emotional prosody, suggesting that only the left amygdala plays a causal role in auditory cortex activation for this type of input (Frühholz et al., 2015). Given the association of the left amygdala with controlled, detailed evaluation of emotional stimuli including language (Phelps et al., 2001; Olsson and Phelps, 2004; Costafreda et al., 2008; Sergerie et al., 2008), this latter result is consistent with slower cortical processing of speech emotional prosody.

In terms of neural temporal course, the processing of emotional speech has been shown to diverge from that of neutral speech around 200 ms after word onset (Schirmer and Kotz, 2006; Paulmann and Kotz, 2008; Paulmann and Pell, 2010). This time range is similar to that described for emotional written words (Kissler et al., 2006; Schacht and Sommer, 2009; Scott et al., 2009; Hinojosa et al., 2010; Citron et al., 2011) and considered to reflect lexical processing in non-primary cortex (Schendan et al., 1998; Cohen et al., 2000a; Grossi and Coch, 2005). A differentiation between emotional categories (e.g., anger, disgust, fear, etc…) based on emotional prosody occurs later, around 300–400 ms (Paulmann and Pell, 2010), and with a different latency for different categories (Pell and Kotz, 2011).

Neurons across auditory cortical fields have differential spectrotemporal response properties that are consistent with the existence of separate processing streams for low- and high- spectral bands in complex sounds. In the core region of primate auditory cortex, neurons in anterior area R integrate over longer time windows than neurons in area A1 (Bendor and Wang, 2008; Scott et al., 2011), and neurons in the lateral belt have preferential tuning to sounds with wide spectral bandwidths compared to the more narrowly-tuned neurons in the core (Rauschecker et al., 1995; Rauschecker and Tian, 2004; Recanzone, 2008). Thus, a posterior-anterior auditory ventral stream from the core is thought to process sounds at increasing longer time scales, and a medial-lateral auditory ventral stream from the core processes sounds at increasing larger spectral bandwidth (Rauschecker et al., 1995; Bendor and Wang, 2008; Rauschecker and Scott, 2009). Indeed, more anterior areas in the superior temporal cortex show sensitivity to increasingly longer chunks of speech (DeWitt and Rauschecker, 2012). Anterior and middle areas of the superior temporal gyrus and sulcus (STG/S) show sensitivity to voice prosody (Kotz et al., 2003; Belin et al., 2004; Humphries et al., 2014) and voice emotional cues (Grandjean et al., 2005; Schirmer and Kotz, 2006), which tend to be slow-varying. In contrast, the middle STG/S is thought to be specifically tuned to the faster spectral transitions relevant to phonemic perception (Liebenthal et al., 2005, 2010, 2014; Obleser et al., 2007; DeWitt and Rauschecker, 2012; Humphries et al., 2014), and more posterior areas in STG/S are important for phonological processing (Wise et al., 2001; Buchsbaum et al., 2005; Hickok and Poeppel, 2007; Chang et al., 2010; Liebenthal et al., 2010, 2013).

In addition to differences in spectrotemporal response properties within auditory cortex in each hemisphere there are differences between the two hemispheres. The right hemisphere has been suggested to be more sensitive to fine spectral details over relatively long time scales and the left hemisphere more sensitive to brief spectral changes (Zatorre and Belin, 2001; Boemio et al., 2005; Poeppel et al., 2008). A related theory is that resting state oscillatory properties of neurons predispose the left auditory cortex for processing at short time scales relevant to the rate of phonemes (gamma band) and the right auditory cortex for processing at longer time scales relevant to the rate of syllables (theta band) (Giraud et al., 2007; Giraud and Poeppel, 2012). Such differences in auditory cortex spectrotemporal sensitivity have been suggested as the basis for the common fMRI finding of right hemisphere dominance for emotional prosody perception, and left hemisphere dominance for speech comprehension (Mitchell et al., 2003; Grandjean et al., 2005). However, whether lateralization differences originate in auditory cortex or result from lateralized feedback connections to auditory cortex cannot be determined without examining the neural dynamics of the involved functional networks. A simultaneous fMRI/ERP study (Liebenthal et al., 2013) found that left lateralization during phonological processing of ambiguous speech syllables occurred early (120 ms) in inferior parietal and ventral central sulcus areas, and only later (380 ms) in the superior temporal gyrus, consistent with left lateralized feedback projections from articulatory somatomotor areas to auditory cortex. Attention to the spectral (non-linguistic) properties of the same sounds elicited early right lateralized activity in homologous parietal regions and bilateral activity in superior temporal gyrus. Drawing from these findings, we suggest that differences in the lateralization of auditory cortex responses to emotional prosody cues in speech could result from hemispheric differences in higher order areas feeding back to auditory cortex rather than from inherent hemispheric differences in auditory cortex spectrotemporal resolution.

In summary, while it appears that short, familiar non-verbal vocalizations can be processed quickly via a subcortical route similar to that described for emotional faces, there is no evidence to date that emotional prosody in speech confers the same advantage. Emotional prosody evolves on longer (suprasegmental) time scales and is conveyed by fine-grained spectral cues. Emotional prosody input may therefore reach the amygdala primarily via slower, indirect routes from auditory and association cortices. There is also no evidence that the verbal information in speech can be processed via a fast route to the amygdala (except, as for written words, possibly for highly-familiar, frequent and salient spoken-words).

## Conclusion

In conclusion, a review of current literature on emotion perception suggests that a fast route to the amygdala akin to that described for facial expressions may also function for other classes of non-linguistic stimuli such as brief emotional non-verbal vocalizations. Although, whether afferent access to the amygdala is specialized for visual biological input and in particular faces, and “borrowed” for auditory (and other sensory) input, is unclear. For language, current evidence points to the importance of lexico-semantic (non-sensory), and perhaps contextual top-down, processing for prioritized perception of emotions. However, fast afferent processing may apply under specific circumstances to highly-familiar and emotionally-salient words. One of the primary challenges in future work will be to determine the neural dynamics of amygdala activation for various types of inputs and under different conditions. Another challenge will be to determine the importance of different parallel routes for emotion processing. These issues should be addressed with methods that can provide both high temporal and high spatial resolution of neural activity in order to identify the time course of activation of the amygdala. Future work will also benefit from taking into account individual differences in the perception of stimulus valence, arousal, and familiarity. In particular, negative or inconclusive findings with respect to amygdala involvement in word and voice perception may in some cases be related to weaker emotional charge and personal relevance of the stimulus material. Future work on the neural dynamics of emotion perception will contribute to our understanding of anxiety and other emotional disorders, as well as help identify the neural circuits that should be targeted for effective training and rehabilitation of disorders which affect emotional function. An important issue will be to identify potent classes of stimuli for direct and indirect activation of the amygdala. In particular, indirect routes may be controlled by fronto-parietal executive circuits that are more amenable to training (for example, Kreifelts et al., 2013; Cohen et al., 2016), whereas direct routes may be less amenable to executive control and training. Another potential implication of research on subcortical routes for perception will be to understand the neural basis of hallucinations. In patients with schizophrenia and auditory or verbal hallucinations, subcortical structures including the thalamus and hippocampus have been proposed to play an important role in generating salient and emotionally charged sensations, whereas the cortical structures with which they are interconnected have been suggested to supply the detailed sensory content of hallucinations (Silbersweig et al., 1995; Silbersweig and Stern, 1998). Resolving the neural dynamics of emotion perception will contribute to our understanding of hallucinations and how they can be treated.

## Author contributions

EL wrote the review, with substantial intellectual input from DS and ES.

### Conflict of interest statement

The authors declare that the research was conducted in the absence of any commercial or financial relationships that could be construed as a potential conflict of interest.
